# Editorial: Circadian rhythm sleep-wake disorders: Pathophysiology, comorbidity, and management

**DOI:** 10.3389/fpsyt.2023.1134798

**Published:** 2023-01-26

**Authors:** Tsuyoshi Kitajima, Kenichi Kuriyama

**Affiliations:** ^1^Department of Psychiatry, Fujita Health University School of Medicine, Toyoake, Japan; ^2^Department of Sleep-Wake Disorders, National Institute of Mental Health, National Center of Neurology and Psychiatry, Tokyo, Japan

**Keywords:** circadian rhythm sleep-wake disorders, circadian disruption, delayed sleep-wake phase disorder, comorbidity, depression, melatonin, melatonin receptor agonist

Circadian rhythm sleep-wake disorders (CRSWDs) are not stand-alone entities—they are often accompanied by various neuropsychiatric phenomena, ranging from minor symptoms to major psychiatric or neurological disorders. More often, circadian disruptions can be hidden players in various disorders, including psychiatric and physical ones. Recent studies have revealed that circadian disruptions/disorders and other disorders have a bidirectional relationship, suggesting some shared pathophysiology.

In this context, the focus of this Research Topic has been on CRSWDs and other conditions related to the circadian system. Otsuki et al. conducted a retrospective study to examine the relationship between delayed sleep-wake phase disorder (DSWPD) and decreased social zeitgebers during the pandemic in Japan. They found that symptoms of DSWPD, as assessed by the clinical global impressions—severity of illness, were worse after the pandemic began, suggesting that not only biological clock regulation in the narrow sense but also environmental factors have substantial contribution in this condition. Tomishima et al. conducted a web survey regarding the epidemiology of the risk of DSWPD. Their results showed that it has a similar prevalence as previous studies in Western countries. Interestingly, this was determined using the Biological Rhythms Interview of Assessment in Neuropsychiatry (BRIAN), originally developed to assess circadian-related symptoms in bipolar disorder (1). The same authors have previously validated BRIAN for the screening of DSWPD in their study (2), and their studies suggest that the phenotype of DSWPD is characterized by neuropsychiatric symptoms besides the deviation of the sleep-wake phase itself. Takaesu et al. studied major depressive disorder (MDD) in terms of the delayed sleep-wake phase and found that it was associated with functional impairments and a deterioration in the quality of life. Their results might lead to important considerations regarding the source of circadian disturbance in MDD. Circadian misalignment may be at the upstream in etiology and bring cognitive and social dysfunction, or it may be the result of weak social zeitgeber (as Otsuki et al. reported), or a third hypothesis would be that the alteration of homeostatic building up in MDD (3) might be the root of both of them. Hirose et al. reported a study of locomotor activities of DSWPD and found that it was altered at the ultradian level compared to healthy people. This is similar to MDD, as shown in the previous study (4), and suggests that DSWPD is not a simple circadian phase disorder. This might also bring some suggestion as to the recent findings that a substantial portion of patients with DSWPD do not have delayed melatonin rhythm, and thus, behavioral components should be weighed in this condition (5).

Besides CRSWDs, Li et al. studied the relationship between neuropsychiatric symptoms and sleep quality in people with cerebral small vessel disease (CSVD). They found a significant association between these factors, which suggests that brain-organic changes may be at the root of both and that there may be shared alteration in neuropsychiatric homeostatic processes. Interestingly, CSVD has also been reported to have relevance to the circadian rhythms of rest-activity (6, 7) or blood pressure (8). This might have another way of association with the alteration of neuropsychiatric homeostasis. This area of research may be worthy of further study in the future. Ichba et al. studied the effect of periocular skin warming on the sleep of patients with insomnia disorder. They reported that the intervention could shorten the sleep onset latency and promote heat dissipation on distal limbs. It is well-known that thermoregulation is under circadian control, and a previous study reported that experimental circadian interventions shortened sleep onset latency in healthy subjects *via* warming distal limbs (9). The present study suggested that this process could be manipulated afferently (i.e., from the peripheral side) in a sophisticated manner, which is interesting for both clinical and etiological considerations.

The melatoninergic function plays an essential role in the central circadian regulatory mechanism, and exogenous melatonin has been used to treat CRSWDs. Palagini et al. reviewed the previous studies on melatonin usage for a broad range of psychiatric conditions and made expert consensus recommendations. Although the evidence so far is still insufficient to cover these conditions fully, it is encouraging that exogenous melatonin seems a promising intervention for their comorbid sleep disorders. The effects of melatonin or ramelteon, a selective MT1 and MT2 receptor agonist, on the symptoms of various psychiatric conditions, have been studied (10), and a recent meta-analysis showed that ramelteon has a preventative effect for relapse of depression in bipolar disorder (11). Melatonin has chronobiotic and soporific properties (10), and whether melatonin receptor agonists work for these conditions *via* direct circadian manipulation (i.e., phase shifting or stabilizing) or not should be investigated further. Polymeropoulos et al. reported that tasimelteon, another selective MT1 and MT2 receptor agonist, could alleviate jet lag disorder in real-life transmeridian travel. Tasimelteon has been reported as a potent compound in circadian modification and approved for treating non-24-h sleep-wake rhythm disorder in the US and Europe (12). The result of the present study is also encouraging because this latest melatonin receptor agonist might potentially expand its usage. There have also been several studies regarding the therapeutic effects of tasimelteon on other conditions, including depression (13). Further studies might also be warranted on its efficacy on CRSWDs' comorbidities.

The articles on this Research Topic have added interesting knowledge to sleep and psychiatric disorders in the context of circadian mechanisms. These should provide further insight into this, a little, complicated theme and inspire researchers to continue exploring it.

## Author contributions

All authors listed have made a substantial, direct, and intellectual contribution to the work and approved it for publication.

## References

[1] Giglio LM Magalhaes PV Andreazza AC Walz JC Jakobson L Rucci P. Development and use of a biological rhythm interview. J Affect Disord. (2009) 118:161–5. 10.1016/j.jad.2009.01.018 19232743

[2] Kanda Y Takaesu Y Kobayashi M Komada Y Futenma K Okajima I. Reliability and validity of the Japanese version of the Biological Rhythms Interview of assessment in neuropsychiatry-self report for delayed sleep-wake phase disorder. Sleep Med. (2021) 81:288–93. 10.1016/j.sleep.2021.02.009 33743475

[3] Borbely AA Daan S Wirz-Justice A Deboer T. The two-process model of sleep regulation: a reappraisal. J Sleep Res. (2016) 25:131–43. 10.1111/jsr.12371 26762182

[4] Nakamura T Kiyono K Yoshiuchi K Nakahara R Struzik ZR Yamamoto Y. Universal scaling law in human behavioral organization. Phys Rev Lett. (2007) 99:138103. 10.1103/PhysRevLett.99.138103 17930642

[5] Duffy JF Abbott SM Burgess HJ Crowley SJ Emens JS Epstein LJ. Workshop report. Circadian rhythm sleep-wake disorders: gaps and opportunities. Sleep. (2021) 44:zsaa281. 10.1093/sleep/zsaa281 33582815 PMC8120340

[6] Sommer R Yu L Schneider JA Bennett DA Buchman AS Lim ASP. Disrupted rest-activity rhythms and cerebral small vessel disease pathology in older adults. Stroke. (2021) 52:2427–31. 10.1161/STROKEAHA.120.030870 33902300 PMC8790726

[7] Zuurbier LA Ikram MA Luik AI Hofman A Van Someren EJ Vernooij MW. Cerebral small vessel disease is related to disturbed 24-h activity rhythms: a population-based study. Eur J Neurol. (2015) 22:1482–7. 10.1111/ene.12775 26206535

[8] Chokesuwattanaskul A Cheungpasitporn W Thongprayoon C Vallabhajosyula S Bathini T Mao MA. Impact of circadian blood pressure pattern on silent cerebral small vessel disease: a systematic review and meta-analysis. J Am Heart Assoc. (2020) 9:e016299. 10.1161/JAHA.119.016299 32476573 PMC7429026

[9] Krauchi K Cajochen C Werth E Wirz-Justice A. Warm feet promote the rapid onset of sleep. Nature. (1999) 401:36–7. 10.1038/43366 10485703

[10] Geoffroy PA Micoulaud Franchi JA Lopez R Schroder CM Membres du Consensus Melatonine SFRMS. The use of melatonin in adult psychiatric disorders: Expert recommendations by the French institute of medical research on sleep (SFRMS). Encephale. (2019) 45:413–23. 10.1016/j.encep.2019.04.068 31248601

[11] Kishi T Nomura I Sakuma K Kitajima T Mishima K Iwata N. Melatonin receptor agonists-ramelteon and melatonin-for bipolar disorder: a systematic review and meta-analysis of double-blind, randomized, placebo-controlled trials. Neuropsychiatr Dis Treat. (2019) 15:1479–86. 10.2147/NDT.S198899 31239683 PMC6553999

[12] Nishimon S Nishino N Nishino S. Advances in the pharmacological management of non-24-h sleep-wake disorder. Expert Opin Pharmacother. (2021) 22:1039–49. 10.1080/14656566.2021.1876665 33618599

[13] Wang YQ Jiang YJ Zou MS Liu J Zhao HQ Wang YH. Antidepressant actions of melatonin and melatonin receptor agonist: Focus on pathophysiology and treatment. Behav Brain Res. (2022) 420:113724. 10.1016/j.bbr.2021.113724 34929236

